# Body Composition, Nutritional Profile and Muscular Fitness Affect Bone Health in a Sample of Schoolchildren from Colombia: The Fuprecol Study

**DOI:** 10.3390/nu9020106

**Published:** 2017-02-03

**Authors:** Mónica Adriana Forero-Bogotá, Mónica Liliana Ojeda-Pardo, Antonio García-Hermoso, Jorge Enrique Correa-Bautista, Emilio González-Jiménez, Jacqueline Schmidt-RíoValle, Carmen Flores Navarro-Pérez, Luis Gracia-Marco, Dimitris Vlachopoulos, Javier Martínez-Torres, Robinson Ramírez-Vélez

**Affiliations:** 1 Centro de Estudios para la Medición de la Actividad Física (CEMA), Escuela de Medicina y Ciencias de la Salud, Universidad del Rosario, Bogota DC 111221, Colombia; monicafore@gmail.com (M.A.F.-B.); monica.ojeda.pardo.1@gmail.com (M.L.O.-P.); jorge.correa@urosario.edu.co (J.E.C.-B.); 2 Laboratorio de Ciencias de la Actividad Física, el Deporte y la Salud, Universidad de Santiago de Chile, USACH, Región Metropolitana, Santiago 7500618, Chile; antonio.garcia.h@usach.cl; 3 Departamento de Enfermería, Facultad de Ciencias de la Salud Avda, De la Ilustración, s/n, (18016), Universidad de Granada, Granada 18071, Spain; emigoji@ugr.es (E.G.-J.); jschmidt@ugr.es (J.S.-R.); carmenf@ugr.es (C.F.N.-P.); 4 Grupo CTS-436, Adscrito al Centro de Investigación Mente, Cerebro y Comportamiento (CIMCYC), Universidad de Granada, Granada 18071, Spain; 5 Children’s Health and Exercise Research Centre (CHERC), Sport and Health Sciences, University of Exeter, Exeter EX1 2LU, UK; L.A.Gracia-Marco@exeter.ac.uk (L.G.-M.); dv231@exeter.ac.uk (D.V.); 6 GENUD “Growth, Exercise, Nutrition and Development” Research Group, University of Zaragoza, Zaragoza 50009, Spain; 7 Grupo GICAEDS, Facultad de Cultura Física, Deporte y Recreación, Universidad Santo Tomás, Bogotá DC 111221, Colombia; javiermartinezt@usantotomas.edu.co

**Keywords:** calcaneal ultrasound, bone health, body fat, muscular strength, calcium

## Abstract

The objective of the present study is to investigate the relationships between body composition, nutritional profile, muscular fitness (MF) and bone health in a sample of children and adolescents from Colombia. Participants included 1118 children and adolescents (54.6% girls). Calcaneal broadband ultrasound attenuation (c-BUA) was obtained as a marker of bone health. Body composition (fat mass and lean mass) was assessed using bioelectrical impedance analysis. Furthermore height, weight, waist circumference and Tanner stage were measured and body mass index (BMI) was calculated. Standing long-jump (SLJ) and isometric handgrip dynamometry were used respectively as indicators of lower and upper body muscular fitness. A muscular index score was also computed by summing up the standardised values of both SLJ and handgrip strength. Dietary intake and degree of adherence to the Mediterranean diet were assessed by a 7-day recall questionnaire for food frequency and the Kidmed questionnaire. Poor bone health was considered using a *z*-score cut off of ≤−1.5 standard deviation. Once the results were adjusted for age and Tanner stage, the predisposing factors of having a c-BUA z-score ≤−1.5 standard deviation included being underweight or obese, having an unhealthy lean mass, having an unhealthy fat mass, SLJ performance, handgrip performance, and unhealthy muscular index score. In conclusion, body composition (fat mass and lean body mass) and MF both influenced bone health in a sample of children and adolescents from Colombia. Thus promoting strength adaptation and preservation in Colombian youth will help to improve bone health, an important protective factor against osteoporosis in later life.

## 1. Introduction 

Quantitative ultrasound (QUS) measurements are widely used to assess bone (especially calcaneal) health because the measurements are non-invasive and both less expensive, and simpler than laboratory-based techniques (i.e., Dual-Energy X-ray Absorptiometry, DXA). However experience and standardization are required to achieve precise measurement [1]. Ultrasound has special appeal for use in youth because of its speed, low cost, and complete lack of ionizing radiation [1]. In recent years, c-BUA at the calcaneus site measured by QUS has been used to determine bone health status in adults [2,3], children [4] and adolescents [5]. Epidemiological evidence shows that peak bone health acquired through bone mineral accrual during childhood and adolescence may be a key determinant of bone health and future fracture risk during a person’s lifespan [6]. It is well known that childhood and adolescence are crucial periods for the development of the skeleton [7]. However, bone quality is determined by a number of factors such as genetic-ethnic factors, body composition, and hormonal status as well as lifestyle behaviours, such as physical activity and diet, that influence bone gain during growth and bone loss later in life [8]. Changes in ultrasound measurements have been shown to be correlated with changes in bone mineral density (BMD) in children and adolescents, and can discriminate between a normal and low BMD [1,2,3,4,5]. A number of studies have also reported QUS parameters to be significantly associated with bone structure independently of BMD [1,2,3,9,10].

Among healthy children and adolescents, bone mass was positively influenced by certain measures of physical fitness as well as by age, weight, and pubertal stage [11]. Previous systematic reviews [12], cross-sectional studies [13,14], and experimental trials [15,16] have examined the influence of body composition, muscular fitness (MF), and nutritional profile, on bone health in children and adolescents, showing controversial findings. Body composition has been implicated in the accrual of bone mass and bone health in children and adolescents [17,18]. Scientific evidence has addressed the relationship between c-BUA with anthropometric and body composition variables such as weight, BMI, and lean mass, even in the long-term [19,20].

Regarding physical fitness, few previous studies have reported on the relationship between MF and c-BUA in youth. Studies based on DXA measurements reported controversial results. Torres-Costoso et al. [13] showed a positive association between upper-limb MF and bone health in schoolchildren, while Cole et al. [21] observed a negative association with volumetric bone density independent of lean mass. Similarly, Gracia-Marco et al. [16] found a positive relationship between MF, speed/agility and cardiorespiratory fitness, and accrual of total-body bone mineral content in 373 adolescents, while Vicente-Rodriguez found in a study of 278 Spanish adolescents that lean mass and MF are an independent predictor of bone health in youth populations [18,22].

On the other hand, healthy diet is an important modifiable factor in the development of bone mass during the critical periods of growth and maturation [23]. In this vein, Tylavsky et al. [24] reported relationships between high fruit and vegetable intakes and bone size/radius in the whole body in early-pubertal white girls after controlling for age, BMI, and physical activity. In addition, calcium intake is considered to be the most important nutrient for bone health throughout a person’s lifespan [25], yet previous cross-sectional and longitudinal studies using QUS have yielded variable results, with a positive association found in some [17,26] but not in others [27]. For this reason, bone measurements are important, even at younger age, to detect a ‘lagged behind’ bone formation at an early stage or, at an older age, to detect a risk profile in time.

In the Latin-American region, most research on QUS has been conducted mainly on adults [28,29], while studies with children and adolescents are scarce [4,30]. At the local level, we have previously reported on body composition correlates of heel QUS among children and adolescents [4], but there is currently no relevant information regarding body composition, MF, and nutritional profile bone gain during growth.

Therefore, the objective of the present study was to investigate the relationship between bone health, body composition, MF, and nutritional profile on a sample of children and adolescents from Colombia.

## 2. Methods

### 2.1. Study Desing and Sample Population 

We performed cross-sectional analyses of baseline data from participants in The FUPRECOL study, which focused on the associations between fitness, health and non-communicable diseases. We have recently published a complete description of The FUPRECOL Study design, methods, and primary outcomes for our current cohort [4,31,32]. In this study, we included a sub-sample (*n* = 1118) of 9 to 17.9 years old healthy Colombian children and adolescents (boys *n* = 508 and girls *n* = 610). The participants were recruited between April 2015 and June 2015 [32]. Individuals with endocrine disorders, psychiatric disorders, pregnancy, cardiovascular disease, obesity, systemic infections, asthma, or other physical impairments that made them unable to participate in this study, as well as those using any prescribed drugs or actively using illegal or illicit drugs, were excluded from this investigation.

### 2.2. Data Collection

All data were collected at the same time in the morning, between 7:00 a.m. and 10:00 a.m. Body weight and height were measured following standard procedures and using an electronic scale (Tanita^®^ BC544, Tokyo, Japan) and a mechanical stadiometer platform (Seca^®^ 274, Hamburg, Germany), respectively. BMI was calculated as body weight in kilograms divided by the square of height in metres. BMI was classified as underweight, normal weight, overweight, or obese using the International Obesity Task Force (IOTF) criteria [33]. Waist circumference (WC) was measured at the midpoint between the last rib and the iliac crest using a tape measure (Ohaus^®^ 8004-MA, Parsippany, NJ, USA). In all measures, we found almost excellent test-retest reliability [body weight (intraclass correlation, ICC = 0.983), height (ICC = 0.973), BMI (ICC 0.897), and WC (ICC = 0.967)]. To classify WC, we used criterion-referenced health-related cut-points derived from de Ferranti et al. [34] because of its large sample size, age-specificity, and relatively generalizable ethnicity. In addition, we also calculated the waist-to-height ratio (WHtR). Ashwel et al. [35] proposed a universal WHtR cutoff >0.5, that might identify early cardiovascular risk in children and adolescents. All cut-off values were based on data obtained from schoolchildren internationally [36,37,38]. Lean mass (kg) and body fat (percentage) were determined for bioelectrical impedance analysis (BIA) by Tanita BC-418^®^. A detailed description of BIA technique can be found elsewhere [39]. The corresponding intra-observer technical error (reliability) of the measurements was 0.95%. Ramírez-Vélez et al. [39] proposed a surrogate cut-off to unhealthy levels of body fatness (24% in boys and 26% in girls) to identify individuals at risk of excess adiposity. Abnormal body compositions such as high body fatness, low lean mass, or a combination of the two phenotypes are relevant indices, but data on their prevalence in youth populations are still limited. In our study, we divided participants into subgroups as proposed by Siervo et al. [37]: healthy lean mass, (upper 50th percentile) and healthy body fatness (≤24% in boys and ≤26% in girls) and unhealthy lean mass (lower 50th percentile) and overfat (>24.1% in boys and >26.1% in girls).

The MF protocols used are appropriate for use in this age group and have shown acceptable validity and reliability [36,40]. We used standing long-jump (SLJ) and isometric handgrip dynamometry as indicators of lower and upper body MF, respectively. To assess lower body MF, subjects were instructed to jump as far as possible using a two footed take-off and landing technique. They were encouraged to flex then extend their knees, ankles, and hips and to swing their arms to maximise performance. SLJ performance was calculated as the distance between the toes at take-off to the heels at the landing point. The best score from two correctly performed jumps was used [40]. Handgrip strength was assessed as an indicator of upper-body MF using an adjustable analogue handgrip dynamometer T-18 TKK SMEDLY III^®^ (Takei Scientific Instruments Co., Ltd, Niigata, Japan). Students watched a brief demonstration of technique and were given verbal instructions on how to perform the test. The dynamometer was adjusted according to the child’s hand size according to predetermined protocols [40]. Monthly, each dynamometer was tested using a standardized calibration procedure which showed that the device was within 1 kg of accuracy over the whole measuring range (from 0 to 100 kg), and with a 100 g sensitivity [41]. SLJ and handgrip measurements in a subsample (*n* = 229, similar in demographics and biological characteristics to the whole sample) were recorded to ensure reproducibility on the day of the study. The reproducibility of our data were *r* = 0.78 to SLJ and *r* = 0.96 to handgrip test. Rather than relying on performance-based normative values, we categorized MF data using cut-off points shown to have cardio-metabolic health predictive values [42]. A muscular index score was computed by summing up the standardized values of SLJ and handgrip strength. The score was calculated separately for boys and girls and by 1-year age groups [38]. To date, there are no established reference cut-off points for MF, assessed using either handgrip strength or SLJ. Previous studies have shown that participants with low MF (lowest quartile) have the poorest cardio-metabolic profile [43,44]; therefore, suggesting that participants falling below the threshold for this quartile could be considered “unhealthy”. Taking this into account, we have used the 20^th^ percentile as a threshold for unhealthy MF, as reported in adolescents from Europe [40].

Food consumption was assessed by the Kidmed questionnaire [45,46]. In this study, we divided participants into two groups: less or equal to 8 points (ideal healthy diet), and less or equal to 7 points (non-ideal healthy diet). In order to obtain information related to calcium-containing foods and soda, we used a seven-day recall. Participants were asked this specific question: “over the past 7 days, on average how many servings of food such as cheese, yogurt, milk, and calcium-fortified orange juice did you have per day?” (or per week if less frequent). Researchers helped participants to better estimate serving sizes (e.g., one cup of milk, one slice of cheese). Soda consumption and its type, in cups per day, was also collected. The 7-day recall test-retest reliability was 0.68. Calcium-containing foods and soda data were analysed with the Nutritionist-diet analysis software (ICBF, Bogota, Colombia), amended to include traditional Colombian recipes according to the Kidmed values validated by Flores Navarro-Pérez et al. [46]. Based on the NIH Consensus Development Program we used 1200 mg/day as optimal calcium intake in children and adolescents [47]. A registered nutritionist conducted the dietary assessment.

c-BUA is dependent on the commercial ultrasound densitometer used. Mathematical adjustments were made to the true c-BUA according to Jaworski et al, [48] and Ramírez-Vélez et al. [4] who used the Achilles ultrasound densitometer (Lunar Corporation, Madison, WI, USA) to measure the c-BUA values in young individuals [3]. A detailed description of c-BUA technique has been described elsewhere [4]. The coefficient of variation (CV) for within-day measurements has previously been reported as 1.8% for c-BUA. The CV for between-day measurements is 0.69 [49]. Poor bone health was considered using a z-score cut off of ≤−1.5 standard deviation, as suggested in clinical practice [50].

Maturation status (self-reported) was assessed by the classification described by Tanner (five stages: I–V) as; pre-pubertal (I–II), pubertal (III), and post-pubertal (IV–V) [51]. Each participant entered into an isolated room where, using a set of images exemplifying the various stages of sexual maturation, they categorized the development of their own genitalia (for boys), breasts (for girls), armpits (for boys) and pubic hair (for both genders). The reproducibility of our data reached 85%.

### 2.3. Ethics Statement

The study protocol was explained verbally to the participants and their parents/guardians before they gave their written consent. Participation in the study was fully voluntary and anonymous, with no incentives provided to participants. The Review Committee for Research on Human Subjects at the University of Rosario (code No. CEI-ABN026-000262) approved all study procedures. The protocol was in accordance with the latest revision of the Declaration of Helsinki and current Colombian laws governing clinical research on human subjects (Resolution 008430/1993 Ministry of Health).

### 2.4. Statistical Analysis 

A power analysis showed that this sample size was sufficient to estimate c-BUA values with a precision of 10% and a power of 90%. The sample size was estimated at 30 participants per age-sex group. Body composition, nutritional profile, sexual maturation, and MF characteristics of the study sample are presented as means, SD or relative frequencies *n* (%). The normality of the variables was verified using histograms and Q-Q plots. Differences were analysed using two-way analysis of variance (ANOVA) or chi-square test (χ^2^) in order to explore sex and age-group (children 9 to 11.9 years vs. adolescents 12 to 17.9 years) differences. Linear regression models and Pearson’s correlation coefficients were used to examine the relationships between c-BUA values and body composition (lean mass and body fat percentage) outcomes by sex and age-group. To estimate the relationship between poor bone health and body composition and anthropometric variables (WC, BMI, WHtR, and age), nutrition profiles (Mediterranean diet quality (low, medium and high), calcium intake (compliance of calcium intake dietary recommendations), sugar-sweetened soft/drink intake (daily, weekly and never)), MF variables (SLJ, handgrip, handgrip/body mass, and muscular index score), and binary logistic regression models were used and adjusted for age and sexual maturation. *Odds ratios* were considered a confounder if they shifted the model in a constant direction with a proportional increase in the exposure level of at least 10%. We used SPSS V. 21.0 software for Windows (SPSS, Chicago, IL, USA). Statistical significance was set at *p* < 0.05.

## 3. Results

### 3.1. Descriptive Characteristics

Table 1 shows the demographic descriptive statistics of the sample. The final sample had a mean age of 13.0 ± 2.3 (range 11–14) years and contained slightly more females (54.6%). Girls had lower levels of weight, z-score BMI, SLJ, handgrip, handgrip/body mass (kg) and muscular index scores than boys (*p* < 0.05). c-BUA average was different by age-group and sex; in girls (children 60.5 (14.8) vs. adolescents 80.8 (15.0) and in boys (children 58.5 (12.1) vs. adolescents 82.9 (18.0) The prevalence of overweight and obesity scores was 23.6% and 10.6% in girls (children 9 to 11.9 years), and 19.9% and 11.2% in boys (*p* < 0.05), according to the IOTF criteria. The average and prevalence of calcium intake foods (portions/day) and compliance with calcium intake daily were 1.5 and 8.7 in boys (adolescents 12 to 17.9 years), and 1.3 and 9.3% in girls, according to the dietary recommendations.

### 3.2. Effects of Anthropometric, Body Composition, Muscular Fitness and Calcium Intake Characteristics on Bone Health (c-BUA) 

Correlations between the subjects’ characteristics (anthropometric, body composition, muscular fitness and calcium intake) for both sexes and the c-BUA values were examined by Pearson correlations (Table 2). In children and adolescents, particularly boys aged 0 to 11.9 years, the c-BUA (dB/MHz) parameter correlated positively with age, weight, height, BMI, WC, fat mass, lean mass, handgrip, SLJ, muscular index score and calcium intake dietary and negatively with WHtR (*r* = −0.141, *p* = 0.048).

### 3.3. Characteristics Associated with Healthy and Poor Bone Health 

The c-BUA ranged from 59.0 to 84.0 dB/MHz (mean 70.3 SD (18.8) dB/MHz). Participants falling within unhealthy lean mass, underweight status, unhealthy muscular index score and handgrip showed a higher poor bone health prevalence (29.6%, 20.7%, 17.9% and 15.5%, respectively). A total of 14.4% of children and adolescents had poor bone health (Table 3).

### 3.4. Factors Associated with Poor Bone Health 

**Figure 1:**
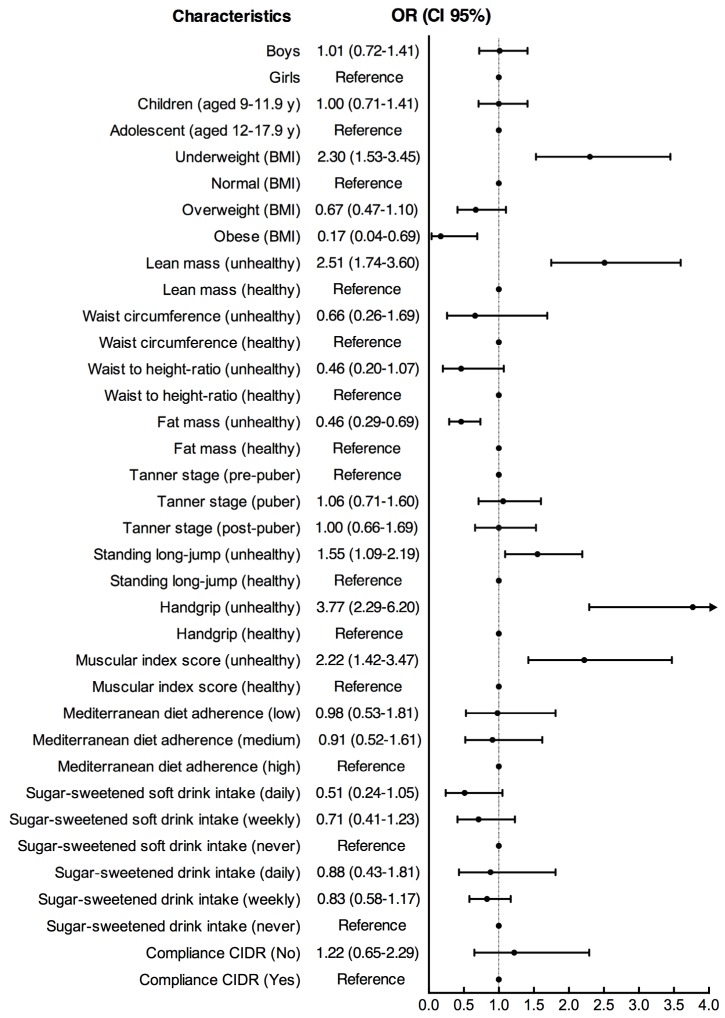
Factors associated with poor bone health.

Figure 1, shows results from the logistic regression analysis. Once the adjustment was performed (by age and Tanner stage), the predisposing factors of having a c-BUA *z*-score ≤−1.5 standard deviation included; being underweight [OR 2.30 (95% CI 1.53 to 1.69)], being obese [OR 0.17 (95% CI 0.04 to 0.69)], having an unhealthy lean mass [OR 2.51 (95% CI 1.74 to 3.60)], unhealthy levels of fat mass [OR 0.46 (95% CI 0.29 to 0.74)], unhealthy SLJ performance [OR 1.55 (95% CI 1.09 to 2.19)], unhealthy handgrip performance [OR 3.77 (95% CI 2.29 to 6.20)], and unhealthy muscular index score [OR 2.22 (95% CI 1.42 to 3.47)].

## 4. Discussion 

The results obtained in this study presented for the first time the relationship between bone health and body composition, nutritional profile, and MF in a sample of children and adolescents from Colombia. Our results show that poor bone health values were significantly related to BMI, lean body, fat mass, SLJ, handgrip, and muscular index score.

Regarding the c-BUA and body composition, in both genders and age-groups, the c-BUA (dB/MHz) parameter correlated positively with age, weight, height, waist circumference and BMI and negatively with WHtR. Our results are consistent with those from previous studies, demonstrating a strong association between anthropometric variables and body composition with c-BUA [17]. However, logistic regression analysis showed that the strongest positive associations were the weight status (overweight and obesity) or having a low lean mass, while the high fat mass was negatively associated. The associations observed in this study confirm previous findings, which are mainly based on studies in children and adolescents and on cross-sectional data. For example, a study of 245 Spanish children showed a positive correlation between c-BUA and weight, body mass index (BMI), and lean mass [14]. This is further supported by Eliakim et al. [52] and Correa-Rodriguez et al. [53], who showed reduced QUS measures in obese children compared to non-obese peers. In addition, fat mass may also have a pathophysiological effect on bone metabolism. This could suggest that schoolchildren from Bogotá have higher levels of adiposity compared to studies developed in other countries [54,55]. Body fat changes during childhood are ethnic-related, which has also been evident in pre-pubertal children and adolescents just from South America. The latter highlights the importance of our study, which is ethnic-specific [1,4]. As the growth spurt occurs during puberty, the natural process of pubertal development can explain these results as well as changes in body composition [56]. When children and adolescents grow, body fat (proportion and content) changes; this occurs more obviously in the pre-adolescent phases when girls continue to present an increase in fat mass [57,58]. Our results also show that obese and underweight youth were respectively associated with healthy or poor bone health, highlighting the role of body weight and lean mass in bone health. These findings could be due to the lower lean mass developed as consequence of their lower fat mass [7]. The hypothesis that the association between body size and QUS parameter may be parameter-specific has been supported by a study in Japanese adolescents [59].

Regarding the MF, the physiological basis for explaining the association between MF and bone mass remains uncertain. Our logistic regression analyses showed that unhealthy MF (SLJ, handgrip and muscular index score) predicts poor bone health. In this regard, a cross-sectional study of 693 school children found that physical fitness using the SLJ had a significant influence on the QUS parameters in children [60]. Also, a 20-year prospective study reported that childhood fitness levels, particularly in females and in the pre- or early pubertal years, were predictive of adult skeletal status as measured by QUS, explaining up to 8% of the variation in adult bone mass [18]. Therefore, our findings are also supported by studies demonstrating a significant association between MF and bone health using DXA measurements. Regarding the MF of upper-limbs, such as handgrip strength, Torres-Costoso et al. [7] findings showed that schoolchildren with good performance in handgrip test had more bone mineral content and BMD in a number of body regions. However, there are controversial results in relation to lower-limbs MF, showing positive [15], negative [8] or no association [61]. Discrepancies between results might be due to several factors, such as genetics, diet, physical activity or sport participation, skeletal age and hormone levels. In addition, one consistent finding between studies was of the role of lean mass to explain bone-related variables [7]. These results suggest the need to promote physical activity with an emphasis on fitness improvement through skeletal loading, which should constitute a key element in the physical education curriculum.

There is a solid body of evidence for the association of nutritional profile and calcium intake with bone mass, especially in adults, but there is a controversy on whether the influence of diet on bone health is mediated by body composition. Also, it is not known whether diet and body composition are independent predictors of poor bone health or whether the risk for poor bone health involved with obesity is modified by MF. In our study, we did not detect significant effect of Mediterranean diet adherence, sugar-sweetened soft drink or calcium intake on c-BUA. This is also confirmed by the results of the multivariate model. Previously, it has been shown in paediatric population that excessive intake of sugar-sweetened beverages may have several adverse effects on human health such as low bone mineral density [62], hypocalcaemia [63] and bone turnover variables [64]. Some of the nutrients contained in sugar-sweetened beverages, like fructose, caffeine and phosphoric acid, have already been proposed to affect the link of such beverages with poor bone health [24,26,27,28]. However, a clear mechanism was not apparent on the basis of these observational data.

Despite calcium being considered the most important nutrient for bone health throughout a person’s lifespan [48], previous studies using QUS have shown controversial results, with positive [12,27] and no associations [7,8,65]. However, recent reports have shown low calcium intake and dairy product consumption globally, especially in children and adolescents [66,67]. We found that inadequate calcium intake is highly prevalent in Colombian children and adolescents. Failure to observe an effect of calcium on c-BUA in the Colombian population compared to another studies might be explained to the criteria selected to define optimal calcium intake (i.e., 1200 mg/day). For example, in young [68] and adolescent females [23,48], it was reported that those subjects whose calcium intake was greater than 1000 mg/day had higher bone measurements (c-BUA) using calcaneus QUS. In contrast, an Iranian cross-sectional study on calcium intake factors in 9 to 12 years old schoolchildren found that dietary calcium intake was not significantly correlated with serum calcium and other selected biochemical indicators of bone health [69]. In Colombia, according to Velásquez et al. [70], calcium is the most limiting nutrient in the youth diet. On the other hand, previous research conducted in Piedecuesta, Colombian reported the prevalence of hypocalcaemia in approximately 42% of six to twelve years old schoolchildren [71]. The need to encourage children and adolescents to eat more calcium-rich products in order to meet their calcium needs should be emphasized.

This study has some limitations. Firstly the cross-sectional design cannot make cause–effect inferences. Secondly it included participants from only a single region in Colombia; therefore, inferences for all Colombian children and adolescents should be made cautiously. The FUPRECOL study was deployed in collaboration with the Bogotá District Education Department, which only has jurisdiction among public schools. Thirdly we have not considered the potential impact of recognized determinants such as socioeconomic status, metabolic biomarkers, physical activity patterns and ethnic factors that modulate growth and levels of adiposity. However, body fat has been considered as a determinant of bone mass and previous studies shown consistent evidence regarding the association of BMC with fat mass [3,19,72]. Furthermore, we have not adjusted our analyses for a wide range of confounders, including vitamin D intake and/or serum 25-hydroxy vitamin D concentration, social class and cardiorespiratory fitness [7,16,18,19,22,53]. A limitation of BMI as a marker of adiposity relates to the variability in fat mass and fat free mass that result in the same BMI in non-overweight children and adolescents. Because it is a weight-for-height measure, BMI does not distinguish between fat mass and lean body mass. Thus individuals with increased lean mass may also have increased BMI. Another limitation of BMI is that the characterization of adiposity may differ across ethnicity and gender and the cut-off point selection in percentiles at the overweight categories [73,74]. The relationship between BMI and c-BUA was reported for many youth populations [4,6,8,14]. Nevertheless, the role of obesity as a risk factor for unhealthy bone mass remains unclear. Finally, QUS data provide only quantitative information and do not allow assessing qualitative factors contributing to bone fragility. Also we have not analysed from QUS measurement the speed of sound and stiffness index. Despite these limitations, the study also has various strong points that should be highlighted. The results presented, for the first time, the relationship between bone health and body composition (fat mass and lean body), MF, and nutritional profile on a large sample of children and adolescents from Colombia.

## 5. Conclusions

Body composition and MF influence bone health on a large sample of children and adolescents from Colombia. Although poor bone health is a serious consequence of the future risk of osteoporosis, the deterioration of bone health requires increased attention among schoolchildren in Bogotá. Thus, promoting strength adaptation and preservation in Colombian youth will help to maximize bone health, an important protective factor against osteoporosis later in life.

## Figures and Tables

**Table 1:** Characteristics among a sample of children and adolescents from Bogota, Colombia (mean (SD) or frequencies).

Characteristics	Total (*n* = 1118)	Girls (*n* = 610)			Boys (*n* = 508)		
		Children 9–11.9 Years (*n* = 246)	Adolescents 12–17.9 Years (*n* = 364)	*p*-Value	Children 9–11.9 Years (*n* = 246)	Adolescents 12–17.9 Years (*n* = 364)	*p*-Value
Age (years)	13.0 (2.3)	10.6 (1.1)	14.6 (1.3)	<0.001	10.4 (1.2)	14.6 (1.2)	<0.001
Weight (kg)	46.6 (11.7)	37.0 (8.5)	51.0 (8.4)	<0.001	37.4 (9.2)	54.0 (10.4)	<0.001
Height (m)	152.0 (12.4)	141.5 (9.0)	155.2 (6.1)	<0.001	140.7 (10.0)	163.0 (9.6)	<0.001
Body mass index (kg/m^2^)	19.9 (3.2)	18.5 (2.9)	21.2 (3.1)	<0.001	18.5 (2.8)	20.2 (3.0)	<0.001
Lean mass (kg)	34.4 (8.7)	60.4 (14.7)	80.0 (15.0)	<0.001	58.5 (12.0)	82.9 (18.0)	<0.001
Body mass index status (%) *							
Underweight	164 (14.7)	43 (17.5)	53 (14.6)	0.004	14 (7.1)	54 (17.3)	<0.001
Normal weight	658 (58.9)	119 (48.3)	207 (56.9)		121 (61.7)	211 (67.6)	
Overweight	221 (19.8)	58 (23.6)	89 (24.5)		39 (19.9)	35 (11.2)	
Obese	75 (6.7)	26 (10.6)	15 (4.1)		22 (11.2)	12 (3.8)	
Waist circumference (cm)	82.6 (9.3)	75.5 (7.5)	88.0 (7.1)	<0.001	75.5 (7.7)	85.6 (7.6)	<0.001
Waist-to-height ratio	0.426 (0.045)	0.427 (0.046)	0.422 (0.043)	0.189	0.447 (0.045)	0.418 (0.043)	<0.001
Fat mass (%)	21.7 (7.5)	60.4 (14.7)	80.8 (15.0)	<0.001	58.5 (12.0)	82.9 (18.0)	<0.001
Tanner stage *	31.5/35.9/32.6	54.1/33.3/12.6	15.4/40.1/44.5	<0.001	62.8/29.6/7.7	12.8/36.9/50.3	<0.001
Prepuber/Puber/Pospuber (%)							
Standing long-jump (cm)	130.8 (29.2)	107.4 (18.8)	127.5 (21.5)	<0.001	120.9 (21.5)	158.8 (26.2)	<0.001
Handgrip (kg)	22.5 (8.1)	16.0 (4.3)	23.0 (4.2)	<0.001	16.4 (4.5)	30.7 (8.4)	<0.001
Muscular index score	0.0 (0.8)	−0.1 (0.8)	0.0 (0.8)	0.046	−0.2 (0.9)	0.0 (0.8)	0.008
c-BUA (dB/MHz)	73.0 (18.9)	60.5 (14.8)	80.8 (15.0)	<0.001	58.5 (12.1)	82.9 (18.0)	<0.001
Mediterranean diet adherence *	30.4/60.2/9.4	29.7/58.5/11.8	35.2/58.0/6.9	0.068	27.6/60.7/11.7	27.2/63.8/9.0	0.577
Low, Medium, High (%)							
Sugar-sweetened soft drink intake *	13.2/78.4/8.4	14.6/72.4/13.0	11.8/79.4/8.8	0.114	14.3/79.1/6.6	13.1/81.7/5.2	0.705
Daily/Weekly/Never							
Sugar-sweetened drink intake *	6.3/53.7/40.0	5.7/49.6/44.7	5.2/48.4/46.4	0.904	7.7/58.7/33.6	7.1/60.3/32.6	0.930
Daily/Weekly/Never							
Calcium intake foods (portions/day)	1.3 (2.0)	1.1 (1.7)	1.3 (2.0)	0.152	1.0 (1.7)	1.5 (2.4)	0.002
Calcium intake dietary recommendations (portions/day)	3.7 (0.7)	3.3 (0.4)	3.5 (0.0)	<0.001	3.2 (0.5)	4.7 (0.6)	<0.001
Compliance with calcium intake dietary recommendations * (daily, yes)	8.7	8.1	9.3	0.606	8.2	8.7	0.847

Data are shown as mean (SD) or frequencies; Significant between-sex differences (ANOVA one way test or chi-square; * *p* < 0.001); c-BUA, calcaneus quantitative ultrasound parameter; Calcium intake and compliance was measured by 7-day recall test.

**Table 2:** Pearson correlation matrix for c-BUA and anthropometric, body composition, muscular fitness and calcium intake characteristics.

Characteristics	Total (*n* = 1118)		Girls (*n* = 610)				Boys (*n* = 508)			
			Children 9–11.9 Years (*n* = 246)		Adolescents 12–17.9 Years (*n* = 364)		Children 9–11.9 Years (*n* = 246)		Adolescents 12–17.9 Years (*n* = 364)	
	*r*	*p*-Value	*r*	*p*-Value	*r*	*p*-Value	*r*	*p*-Value	*r*	*p*-Value
Age (years)	0.663	<0.001	0.429	<0.001	0.283	<0.001	0.443	<0.001	0.492	<0.001
Weight (kg)	0.700	<0.001	0.550	<0.001	0.484	<0.001	0.630	<0.001	0.535	<0.001
Height (m)	0.649	<0.001	0.449	<0.001	0.310	<0.001	0.631	<0.001	0.443	<0.001
Body mass index (kg/m^2^)	0.480	<0.001	0.416	<0.001	0.376	<0.001	0.417	<0.001	0.352	<0.001
Lean mass (kg)	0.689	<0.001	0.543	<0.001	0.460	<0.001	0.679	<0.001	0.575	<0.001
Waist circumference (cm)	0.475	<0.001	0.328	<0.001	0.404	<0.001	0.409	<0.001	0.347	<0.001
Waist-to-height ratio	0.015	0.624	0.021	0.995	0.085	0.103	−0.141	<0.001	0.018	0.747
Fat mass (%)	0.082	0.006	0.320	<0.001	0.325	<0.001	0.212	0.090	0.044	0.451
Standing long-jump (cm)	0.391	<0.001	0.229	<0.001	0.121	0.020	0.170	0.017	0.199	<0.001
Handgrip (kg)	0.644	<0.001	0.469	<0.001	0.323	<0.001	0.526	<0.001	0.580	<0.001
Muscular index score	0.259	<0.001	0.277	<0.001	0.151	<0.001	0.391	<0.001	0.278	<0.001
Calcium intake foods (portions/day)	0.069	0.021	0.028	0.661	0.016	0.760	0.015	0.829	0.041	0.469
CIDR (portions/day)	0.453	<0.001	0.236	<0.001	0.281	<0.001	0.320	<0.001	0.338	<0.001

*r* = correlation coefficient (Pearson’s *r*); CIDR, calcium intake dietary recommendations.

**Table 3:** Prevalence and anthropometric, body composition, muscular fitness and nutritional profile characteristics associated with healthy and poor bone.

Characteristics	Adequate Bone Healthy (*n* = 956)				Poor Bone Healthy (*n* = 162)				*p*-Value
	*n*	%	CI 95%		*n*	%	CI 95%		
Girls	522	54.6	51.7	57.8	88	54.3	46.3	62.3	0.947
Boys	434	45.4	42.2	48.3	74	45.7	37.7	53.7	
Children (aged 9–11.9 years)	378	39.5	36.5	42.6	64	39.5	31.5	46.9	0.994
Adolescents (aged 12–17.9 years)	578	60.5	57.4	63.5	98	60.5	53.1	68.5	
Underweight (BMI)	119	12.4	10.6	14.4	45	27.8	20.4	34.6	<0.001
Normal (BMI)	565	59.1	56.0	62.1	93	57.4	49.4	64.8	
Overweight (BMI)	199	20.8	18.3	23.4	22	13.6	8.6	19.1	
Obese (BMI)	73	7.6	6.0	9.3	2	1.2	0.0	3.1	
Lean mass (unhealthy)	491	51.4	48.0	54.4	48	29.6	22.8	36.4	
Lean mass (healthy)	465	48.6	45.6	52.0	114	70.4	63.6	77.2	
Waist circumference (unhealthy)	44	4.6	3.3	6.0	5	3.1	0.6	6.2	0.387
Waist circumference (healthy)	912	95.4	94.0	96.7	157	96.9	93.8	99.4	
Waist to height-ratio (unhealthy)	74	7.7	6.2	9.5	6	3.7	1.2	6.8	0.072
Waist to height-ratio (healthy)	882	92.3	90.5	93.8	156	96.3	93.2	98.8	
Fat mass (unhealthy)	252	26.4	23.7	29.1	23	14.2	9.3	20.4	<0.001
Fat mass (healthy)	704	73.6	70.9	76.3	139	85.8	79.6	90.7	
Tanner stage (pre-pubertal)	302	31.6	28.6	34.4	50	30.9	24.1	37.7	0.945
Tanner stage (pubertal)	341	35.7	32.6	38.6	60	37.0	29.6	44.4	
Tanner stage (post-pubertal)	313	32.7	29.8	35.8	52	32.1	25.3	39.5	
Standing long-jump (unhealthy)	526	55.0	52.0	57.9	106	65.4	58.6	72.2	0.014
Standing long-jump (healthy)	430	45.0	42.1	48.0	56	34.6	27.8	41.4	
Handgrip (unhealthy)	637	66.6	63.5	69.7	143	88.3	82.7	93.2	<0.001
Handgrip (healthy)	319	33.4	30.3	36.5	19	11.7	6.8	17.3	
Muscular index score (unhealthy)	92	9.6	7.7	11.5	31	19.1	13.6	25.3	<0.001
Muscular index score (healthy)	864	90.4	88.5	92.3	131	80.9	74.7	86.4	
Mediterranean diet adherence (low)	289	30.2	27.4	33.3	51	31.5	24.7	38.9	0.908
Mediterranean diet adherence (medium)	578	60.5	57.5	63.6	95	58.6	50.6	66	
Mediterranean diet adherence (high)	89	9.3	7.5	11.2	16	9.9	5.6	14.8	
Sugar-sweetened soft drink intake (daily)	132	13.8	11.7	15.9	16	9.9	5.6	14.8	0.186
Sugar-sweetened soft drink intake (weekly)	749	78.3	75.7	81.0	128	79.0	72.2	85.2	
Sugar-sweetened soft drink intake (never)	75	7.8	6.2	9.6	18	11.1	6.8	16	
Sugar-sweetened drink intake (daily)	60	6.3	4.7	7.9	10	6.2	3.1	9.9	0.413
Sugar-sweetened drink intake (weekly)	520	54.4	51.0	57.6	81	50.0	42	57.4	
Sugar-sweetened drink intake (never)	376	39.3	36.3	42.7	71	43.8	36.4	51.8	
Compliance CIDR (No)	871	91.1	89.1	92.9	150	92.6	88.9	96.3	0.536
Compliance CIDR (Yes)	85	8.9	7.1	10.9	12	7.4	3.7	11.1	

BMI, body mass index; CIDR, calcium intake dietary recommendations.

## Abbreviations

The following abbreviations are used in this manuscript:

BIA	bioelectrical impedance analysis
BMC	bone mineral content
BMD	bone mineral density
BMI	body mass index
c-BUA	calcaneus quantitative ultrasound parameter
DXA	dual-energy X-ray absorptiometry
MF	muscular fitness
SLJ	standing long-jump
QUS	quantitative ultrasonography
WHtR	waist-to-height ratio
